# Isolated Mild Fetal Tricuspid Regurgitation in Low-Risk Pregnancies: An Incidental Doppler Finding or a Marker of Postnatal Cardiac Risk?

**DOI:** 10.3390/children12070879

**Published:** 2025-07-03

**Authors:** Akif Kavgacı, Utku Arman Örün, Özkan Kaya, Mehmet Emre Arı

**Affiliations:** Department of Pediatric Cardiology, Ankara Etlik City Hospital, Ankara 06170, Turkey; utkuarman@hotmail.com (U.A.Ö.); drozkankaya@gmail.com (Ö.K.); memreari@yahoo.com (M.E.A.)

**Keywords:** fetal tricuspid regurgitation congenital heart disease, postnatal cardiac outcomes

## Abstract

**Background:** Tricuspid regurgitation (TR) is increasingly recognized as a detectable finding during routine fetal echocardiography. Although previous studies have explored its potential role as an indirect marker for congenital heart disease (CHD) in the first trimester, the prognostic significance of isolated mild TR in chromosomally normal and low-risk fetuses during the second and third trimesters remains unclear. Clarifying the clinical relevance of this commonly encountered Doppler finding is essential to guide appropriate prenatal management and avoid unnecessary interventions in low-risk pregnancies. **Materials and Methods:** This retrospective study reviewed fetal echocardiography reports of 1592 pregnant women referred to a pediatric cardiology clinic after the 20th gestational week between 1 January 2024 and 1 January 2025. Following exclusion criteria, 1072 low-risk pregnancies were included. A total of 136 fetuses with TR were identified, and among them, postnatal echocardiographic outcomes of 60 neonates who underwent transthoracic echocardiography within the first 10 days after birth were analyzed. **Results:** Among the 1072 low-risk pregnancies included in the study, a total of 136 fetuses were diagnosed with TR on fetal echocardiography. The majority of these cases were characterized as mild and isolated, without accompanying structural abnormalities. Postnatal echocardiographic assessments revealed no major congenital cardiac anomalies, reinforcing the interpretation that isolated mild TR in the context of low-risk pregnancies represents a benign and likely transient physiological finding. **Conclusion:** Isolated mild TR, particularly in low-risk and chromosomally normal pregnancies, appears to be a transient and clinically insignificant finding. These results support the interpretation of fetal TR in the context of overall clinical and structural evaluation, helping to avoid unnecessary interventions and reduce parental anxiety.

## 1. Introduction

Congenital heart defects (CHDs) account for approximately one-third of all congenital malformations and represent a leading cause of infant mortality due to structural anomalies [1]. As the most frequently identified congenital abnormalities during the fetal period, CHDs are associated with intrauterine fetal loss in approximately 5% of cases and neonatal mortality in up to 20% [2,3,4,5]. The increasing clinical use of fetal echocardiography, alongside advances in imaging technologies, has substantially enhanced the prenatal detection and assessment of not only major structural cardiac anomalies but also milder defects and hemodynamic alterations.

Fetal tricuspid regurgitation (TR) occurs due to incomplete coaptation of the tricuspid valve leaflets, typically when right ventricular pressure exceeds right atrial pressure during systole. With the evolution of color Doppler ultrasonography, this pathophysiological phenomenon is now more frequently identified in the prenatal setting. This increased recognition is largely attributable to advancements in echocardiographic technology, and it may also be influenced by the ability to capture favorable fetal positioning during the examination, which can enhance the likelihood of detecting tricuspid regurgitation. However, the clinical significance of fetal tricuspid regurgitation as a predictor of postnatal structural cardiac abnormalities including atrial septal defects, ventricular septal defects, and patent ductus arteriosus remains unclear, particularly as most of these anomalies are considered minor and clinically insignificant [6]. While a limited number of studies have explored the relationship between fetal TR and postnatal outcomes, evidence specifically addressing isolated mild TR in structurally normal, chromosomally low-risk fetuses during the second and third trimesters remains scarce. Our study aims to address this gap by evaluating a well-defined low-risk cohort and correlating prenatal TR with postnatal echocardiographic findings.

Given the current limitations in the literature, the primary objective of this study is to determine the prevalence of fetal tricuspid regurgitation in low-risk pregnancies after 20 weeks of gestation, to assess its potential clinical relevance during the prenatal period, and to systematically examine its association with neonatal echocardiographic outcomes.

## 2. Materials and Methods

### 2.1. Study Population

In this retrospective study, fetal echocardiography reports of 1592 pregnant women who were referred to the pediatric cardiology outpatient clinic for fetal echocardiographic evaluation after the 20th week of gestation between 1 January 2024 and 1 January 2025 were reviewed. Based on predefined exclusion criteria, 1072 low-risk pregnancies were selected. Among these, 136 cases diagnosed with fetal tricuspid regurgitation were identified and planned to be included in the study. Additionally, postnatal transthoracic echocardiography (TTE) results of 60 newborns, whose mothers returned for follow-up within the first ten days after delivery, were also planned to be included for postnatal evaluation.

Maternal age, paternal age, gestational age at the time of fetal echocardiography, referring institution and indication for referral, presence and severity of tricuspid regurgitation, diagnoses, gravida–parity–abortion–living status, and postnatal echocardiographic findings of the neonate were recorded.

Cases were excluded from the study if any of the following criteria were present: maternal age over 35 years, multiple gestation, obstetric history of fetal heart disease, diagnosis of fetal cardiopathy, family history of congenital heart disease, presence of maternal and/or fetal chronic disease, diagnosis of fetal congenital malformation and/or chromosomal abnormality, or increased nuchal translucency (NT). In addition, cases with echocardiographic findings suggestive of structural tricuspid valve abnormalities, in particular Ebstein’s anomaly, were excluded based on valve morphology and insertion plane evaluation.

This study was approved by the Scientific Research Evaluation and Ethics Committee of the Ministry of Health Ankara Etlik City Hospital (Approval Number: AEŞH-BADEK2-2025-061, Date: 13 May 2025), and was conducted in accordance with the principles of the Declaration of Helsinki.

### 2.2. Fetal and Neonatal Echocardiography

All fetal echocardiographic examinations were performed by an experienced pediatric cardiologist using a high-frequency transducer (1.5 MHz, GE C1-5 RS) and a GE Voluson S10 ultrasound system (GE Healthcare, Chicago, IL, USA). The fetal heart was visualized in multiple planes, and all measurements were conducted in accordance with the American Institute of Ultrasound in Medicine (AIUM) Practice Guideline for the Performance of Fetal Echocardiography, which was developed by the AIUM in collaboration with the American College of Obstetricians and Gynecologists (ACOG), the Society for Maternal-Fetal Medicine (SMFM), and the American Society of Echocardiography (ASE), and endorsed by the American College of Radiology (ACR) [7].

Tricuspid regurgitation (TR) was assessed using color Doppler imaging in either the apical four-chamber view or the high short-axis view, with an insonation angle between 0° and 30° during ventricular systole. The severity of TR was evaluated based on the length of the regurgitant jet within the right atrium: a jet extending less than one-third of the distance from the tricuspid valve to the opposite atrial wall was classified as mild, between one-third and two-thirds as moderate, and greater than two-thirds as severe [8]. Postnatal echocardiography was performed within the first 10 days after birth using a high-frequency transducer (2.7–12.5 MHz, GE 12S-RS) and the GE Voluson S-10 system (GE Healthcare, USA), by an experienced pediatric cardiologist.

### 2.3. Statistical Analysis

Descriptive statistics were presented as median and interquartile range for continuous variables and frequency and percentage for categorical variables. Independent group comparisons were made using independent sample t-tests and chi-square tests for continuous and categorical variables, respectively. All statistical analyses were performed in SPSS, version 25.0 (IBM Inc., Armonk, NY, USA), and a type-I error level of 5% (*p* < 0.05) was considered as the threshold limit for statistical significance.

## 3. Results

**Figure 1:**
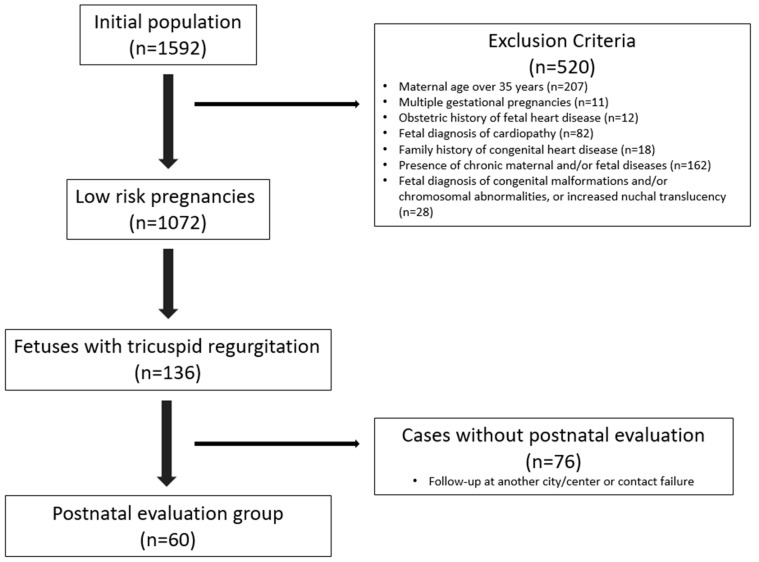
Flowchart of study design and patient inclusion/exclusion criteria.

A total of 2661 fetal ultrasonographic examinations were performed on 1592 singleton pregnancies within the scope of our study. Following the application of exclusion criteria, 1072 eligible participants were included in the final analysis. Among these 1072 pregnancies, fetal tricuspid regurgitation was identified in 136 cases (12.6%). The flowchart of the study cohort selection is presented in Figure 1.

Among the 1072 low-risk singleton pregnancies included in the study, 936 cases without TR on fetal echocardiography had a median maternal age of 29 years (IQR: 25–32), and the median gestational day at the time of fetal echocardiography was 169 days (IQR: 159–187). In contrast, the 136 cases with TR had a median maternal age of 29 years (IQR: 25–33), and TR was detected at a median gestational age of 188.5 days (IQR: 174.5–214.5). The median paternal age was 32 years (IQR: 28–36) in fetuses without TR and 32 years (IQR: 28–37) in those with TR. No statistically significant differences were observed between the groups in terms of maternal age (*p* = 0.116) or paternal age (*p* = 0.908). However, the gestational age at the time of fetal echocardiography was significantly higher in the TR group (*p* < 0.01).

Regarding the severity of regurgitation, 98.5% (*n* = 134) of the cases were classified as mild and 1.5% (*n* = 2) as moderate. Notably, tricuspid regurgitation was detected after the 34th gestational week in seven fetuses. The maternal and obstetric characteristics of the 136 pregnancies with fetal TR are summarized in Table 1.

Postnatal clinical and echocardiographic evaluation was available in 60 out of 136 cases with fetal TR (44.1%). The mean gestational age at birth was 270 days (range: 221–285 days). In the postnatal period, 54 newborns were asymptomatic. Six neonates (10%) were admitted to the neonatal intensive care unit (NICU). The reasons for admission included respiratory distress (*n* = 2), prematurity-related complications (*n* = 2), hypoglycemia (*n* = 1), and hypoxic-ischemic encephalopathy (*n* = 1). All were discharged without complications.

According to postnatal echocardiographic findings, the most frequently observed abnormality was isolated patent foramen ovale (PFO), detected in 63.3% of cases (*n* = 38). This was followed by PFO in combination with left peripheral pulmonary stenosis in 10% (*n* = 6), and PFO with a small patent ductus arteriosus (PDA) in 6.7% (*n* = 4) of cases. A normal echocardiographic finding was found in 5% (*n* = 3) of neonates.

**Figure 2:**
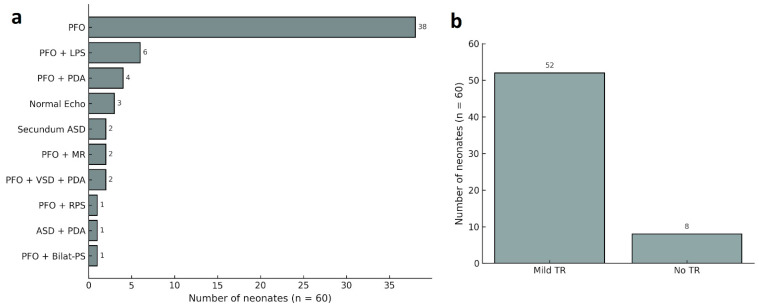
Postnatal echocardiographic characteristics and TR status in fetuses with isolated mild tricuspid regurgitation. (**a**) Distribution of postnatal echocardiographic findings in neonates with prenatally identified tricuspid regurgitation. (**b**) Postnatal tricuspid regurgitation status in the evaluated neonates. Abbreviations: PFO: patent foramen ovale, ASD: atrial septal defect, VSD: ventricular septal defect, PDA: patent ductus arteriosus, LPS: left peripheral pulmonary stenosis, MR: mitral regurgitation, RPS: right peripheral pulmonary stenosis; ASD: atrial septal defect; Bilat-PS: Bilateral peripheral pulmonary stenosis, TR: tricuspid regurgitation.

Less common findings included isolated secundum-type atrial septal defect (ASD) in 3.3% of cases (*n* = 2) and PFO accompanied by mitral regurgitation (MR) in another 3.3% (*n* = 2). Additionally, several rare combinations were identified. These included PFO with right peripheral pulmonary stenosis (1.7%, *n* = 1); secundum ASD with a small PDA (1.7%, *n* = 1); PFO with bilateral peripheral pulmonary stenosis (1.7%, *n* = 1); and PFO with a small PDA and muscular VSD (3.3%, *n* = 2). Mild tricuspid regurgitation was identified in 52 neonates (86.7%), while no regurgitation was observed in the remaining 8 cases (13.3%). None of the cases exhibited hemodynamic significance or required clinical intervention. Among the seven fetuses with tricuspid regurgitation identified after the 34th gestational week, four underwent postnatal echocardiographic evaluation. Of these, three had PFO and one had both PFO and a small PDA. A detailed summary of postnatal echocardiographic findings is provided in Figure 2.

## 4. Discussion

This study evaluated the prevalence of fetal TR in low-risk pregnancies and its association with postnatal cardiac outcomes. The findings indicate that although isolated mild TR was detected during the prenatal period, it was not associated with clinically significant cardiac pathology postnatally. The observation that 98.5% of prenatal TR cases were classified as mild, and that no major cardiac anomalies or need for postnatal intervention were identified, suggests that fetal TR is most often a physiological variant. These results are consistent with findings reported in numerous previous studies.

In the study conducted by Lopes et al., the prevalence of fetal TR in the third trimester was reported to be 10%, with the majority of cases (90.9%) classified as mild. Postnatally, only one newborn exhibited persistent mild TR, and no significant cardiac pathology was identified in any of the patients [9]. While the study by Lopes et al. contributed valuable data on the prevalence and clinical insignificance of fetal tricuspid regurgitation in the third trimester of low-risk pregnancies, it was limited in scope regarding sample size and gestational window. Their analysis focused solely on third-trimester cases and included a relatively small number of postnatal evaluations. In contrast, our study assessed a larger cohort that encompassed both second and third trimesters, thereby enhancing the strength and generalizability of current evidence regarding the benign nature of mild fetal TR in low-risk pregnancies. Zhou et al. followed 86 fetuses diagnosed with fetal TR during the second trimester and reported that none of the cases were associated with major cardiac defects postnatally [6]. These findings are in agreement with the hypothesis that isolated TR may represent a physiological variant in late gestation, and are further supported by our study outcomes, which demonstrated no incidence of major cardiac anomalies in neonates born from low-risk pregnancies.

Clerici et al. reported TR in 4.74% of fetuses among a cohort of 675 pregnancies; notably, no cases of TR were detected beyond the 34th week of gestation [10]. In our study, TR was identified in seven fetuses beyond the 34th week of gestation, four of whom returned for postnatal follow-up. Among these four neonates, PFO was detected in three cases, while one had a PFO and small PDA. These findings suggest that even TR detected after the 34th week of gestation is not associated with major cardiac anomalies in the postnatal period.

In a systematic review conducted by Scala et al., it was emphasized that first-trimester indirect markers including NT, TR, ductus venosus (DV) flow, and abnormal cardiac axis are significantly associated with an increased risk of CHD. The authors noted that while TR observed in high-risk fetuses may have potential value as a screening marker when considered alongside additional findings, the presence of isolated TR was not significantly associated with CHD [5]. In another study evaluating 1075 fetuses during the first trimester, TR was identified in 96 cases. Among the 72 fetuses with aneuploidy, TR was detected in 33.33%, whereas only 6.38% of the 1003 euploid fetuses exhibited TR. When the association between TR and CHD was analyzed within the euploid group, no statistically significant relationship was observed (*p* = 0.06) [11]. Similarly, in a cohort of 486 fetuses examined between 16 and 23 weeks of gestation, TR was identified in 4.3% of cases, with further stratification by karyotype revealing an incidence of 3.2% among euploid fetuses, 50% in those with Down syndrome, and 7.1% in fetuses with other chromosomal anomalies [12]. These findings highlight a stronger association between TR and aneuploidy, particularly trisomy 21. The three aforementioned studies focused on early gestational periods and demonstrated that isolated TR is not statistically significantly associated with major structural cardiac abnormalities in low-risk fetuses. These findings support the notion that mild TR observed during the fetal period in low-risk pregnancies is often transient and clinically insignificant. Although each of these studies focused exclusively on first- or second-trimester screening and demonstrated that isolated TR is not significantly associated with congenital heart defects, their findings were limited to the early gestational period. In contrast, our study extends the clinical context by evaluating isolated mild TR detected during the second and third trimesters in a well-defined, low-risk population. By correlating prenatal findings with postnatal echocardiographic outcomes, we provide evidence that mild, isolated TR observed later in gestation similarly lacks association with major structural cardiac abnormalities. These results support the notion that, across gestation, isolated TR in otherwise low-risk pregnancies is frequently a benign and transient Doppler finding without clinical consequence.

Collectively, these findings suggest that isolated mild TR, particularly in chromosomally normal and low-risk pregnancies, is frequently a transient physiological phenomenon and lacks association with significant postnatal cardiac anomalies. This study highlights the critical importance of interpreting fetal TR within its clinical context, alongside any coexisting structural abnormalities, thereby contributing to a more informed and evidence-based prenatal risk stratification, while also reducing the likelihood of unnecessary clinical interventions.

Building on these findings, our study comprehensively addressed its threefold aim by providing detailed data on the prevalence, clinical significance, and neonatal outcomes associated with fetal TR. The observed prevalence of 12.6% among low-risk pregnancies is consistent with previous reports and further reinforces the understanding that mild fetal TR is not uncommon during the second and third trimesters. In terms of clinical relevance, our findings suggest that isolated mild TR detected in the second and third trimesters is not associated with major structural cardiac anomalies and may reflect a transient hemodynamic variation rather than pathological regurgitation. Finally, neonatal follow-up data confirmed that in the absence of coexisting cardiac findings, mild fetal TR does not adversely affect postnatal cardiac outcomes. Together, these results provide reassurance for clinicians and expectant families, suggesting that isolated mild fetal TR can generally be regarded as a benign prenatal Doppler finding.

## 5. Limitations

The main limitation of this study is that postnatal echocardiographic follow-up data were available for only 60 cases. A larger follow-up cohort could have enhanced the generalizability of the findings. However, this limitation is largely attributable to the nature of our institution as a cardiology referral center, where cases of isolated and clinically insignificant TR detected prenatally are often followed postnatally at local healthcare facilities. As a result, the number of cases with comprehensive postnatal evaluation remained limited. In addition, since our study specifically focused on fetuses with isolated mild TR, systematic postnatal follow-up was not performed for fetuses without this Doppler finding. Among the 936 fetuses without TR who underwent prenatal evaluation at our center, no major structural cardiac abnormalities were identified during fetal echocardiographic assessment. Nevertheless, we acknowledge that inclusion of long-term follow-up data for this broader cohort could have further strengthened the study. This study also did not investigate the association between the degree of fetal TR and right ventricular mechanics. Recent evidence suggests that advanced imaging modalities such as speckle-tracking echocardiography may offer valuable insights into this relationship, particularly in both uncomplicated and complicated pregnancies. Further studies incorporating these techniques could help clarify the functional implications of fetal TR.

## 6. Conclusions

Isolated mild TR identified during the prenatal period appears to be a transient and clinically insignificant finding, particularly in low-risk pregnancies. In our study, postnatal evaluations revealed no major cardiac anomalies and no need for further medical intervention. These findings suggest that fetal TR may largely represent a physiological variant. Therefore, in the absence of associated structural abnormalities, it is important to provide counseling based on clinical necessity, thereby avoiding undue parental anxiety. In this context, our findings support the objective of the study to clarify the clinical implications of isolated mild fetal TR and indicate that routine follow-up is generally sufficient in such cases. Moreover, the accurate interpretation of prenatal cardiac findings necessitates effective multidisciplinary collaboration among obstetricians, neonatologists, and pediatric cardiologists, ensuring that perinatal decision-making processes are guided by an integrated and evidence-based approach.

## Figures and Tables

**Table 1:** Maternal and obstetric characteristics of pregnancies with fetal tricuspid regurgitation identified on echocardiographic examination.

Category	Variable	*n* (%) or Median (IQR)
Maternal Characteristics	Maternal Age (years)	29 (25–33)
	Gestational Age at Diagnosis (days)	188.5 (174.5–214.5)
Obstetric History	Gravida = 1	46 (33.8%)
	Gravida = 2	40 (29.4%)
	Gravida ≥ 3	50 (36.8%)
	Parity = 0	49 (36%)
	Parity ≥ 1	87 (64%)
	Abortus = 0	106 (77.9%)
	Abortus ≥ 1	30 (22.1%)
	Living Children = 0	51 (37.5%)
	Living Children ≥ 1	85 (62.5%)
Referring Department	Obstetrics	62 (45.6%)
	Perinatology	63 (46.3%)
	Other Institutions	11 (8.1%)
Indication for Referral	Suspected CHD	20 (14.7%)
	Suspected TR	5 (3.7%)
	Suboptimal Fetal position	43 (31.6%)
	Echogenic Focus	68 (50%)
Diagnosis	Mild TR	94 (69.1%)
	Moderate TR	2 (1.5%)
	Mild TR with Echogenic Focus	40 (29.4%)

Abbreviations: CHD: congenital heart disease, TR: tricuspid regurgitation.

## Data Availability

No datasets were generated or analyzed during the current study.
